# Robotic radical lymphadenectomy without touching the pancreas during gastrectomy for gastric cancer

**DOI:** 10.1097/MD.0000000000015091

**Published:** 2019-03-15

**Authors:** Toshiyasu Ojima, Masaki Nakamura, Mikihito Nakamori, Keiji Hayata, Masahiro Katsuda, Shimpei Maruoka, Hiroki Yamaue

**Affiliations:** Second Department of Surgery, Wakayama Medical University, Wakayama, Japan.

**Keywords:** gastric cancers, laparoscopic gastrectomy, minimally invasive surgery, pancreatic fistula, robotic gastrectomy

## Abstract

Laparoscopic lymph node dissection around the peripancreatic area for gastric cancer (GC) remains challenging because of drawbacks in laparoscopic surgery including the limited range of movement, amplification of hand tremors, and inconvenient surgical positioning. In some cases of laparoscopic gastrectomy (LG), therefore, postoperative pancreatic fistula (POPF) occurs. Robotic surgery, on the other hand, plays an essential role in ergonomics and offers advantages, such as motion scaling, less fatigue, tremor filtering, 7 degrees of motion in the robotic instruments assisted by the wrist-like instruments tips, and three-dimensional vision. Robotic gastrectomy (RG) may enable surgeons to overcome the drawbacks associated with laparoscopic surgery. This study compares the safety and feasibility of short-term surgical outcomes of RG and LG for patients with GC.

This was a single-center retrospective study of 659 consecutive patients with GC who received minimally invasive surgery. LG (n = 639) was performed between 2013 and 2017 and RG (n = 20) was performed in 2017. Lymphadenectomy without touching the pancreas was basically performed during RG using assisting articulating forceps.

Overall incidence of postoperative complications higher than Clavien–Dindo grade 2 was not significantly different (LG group 5.9%, RG group 5.0%). In RG group, POPF, intra-abdominal abscess, and anastomotic leakage were not found, but postoperative bleeding requiring interventional catheter embolization occurred in 1 patient. In LG, POPF was found in 4.7%. Amylase levels in drainage fluid on postoperative day 1 were significantly lower in the RG group (238.5 IU/L) than in the LG group (884.5 IU/L) (*P* = .028).

Regarding short-term surgical outcomes, RG is feasible, safe, and ideal treatment procedure for GC. Our robotic procedure without touching the pancreas may be associated with decreased incidence of POPF.

## Abstract 2

Supplemental Digital Content is available in the text

## 1 Introduction

Minimally invasive surgery for gastric cancer (GC), typified by laparoscopic gastrectomy (LG), is supported by several studies that demonstrate its safety, feasibility, and oncological suitability compared with open gastrectomy (OG).^[1–4]^

Laparoscopic lymph node dissection around the peripancreatic area, which includes the suprapancreatic and the infrapyloric lymph nodes, however, remains challenging. LG has several drawbacks, including a limited range of movement, amplification of hand tremors, and inconvenient surgical positioning. Notably, postoperative pancreatic fistula (POPF) occurs in around 5% of patients that undergo LG.^[5,6]^ Direct manipulative trauma by pancreatic compression using assisting forceps and/or thermal injury of the pancreas by use of laparoscopic ultrasonically activated device (USAD) may occur during lymph node dissection of the peripancreatic area.

Robotic surgery has ergonomic advantages over conventional laparoscopy, including 7 degrees of motion in the robotic instruments assisted by the wrist-like instruments tips, less fatigue, tremor filtering, motion scaling, and three-dimensional vision.^[7,8]^ Robotic gastrectomy (RG) may overcome some of the drawbacks associated with LG. Since robotic lymphadenectomy that does not come into contact with the pancreas is a possibility, the incidence of POPF may be reduced. However, few studies have assessed the advantages of RG over LG.^[9–11]^ Here, we outline our robotic lymphadenectomy techniques that do not come into contact with the pancreas. We also compare the safety and feasibility of surgical outcomes, including the incidence of POPF by RG and LG in patients with GC.

## 2 Materials and methods

### 2.1 Patients

We performed R0 curative gastrectomy for GC on 785 patients between January 1, 2013, and December 31, 2017, at the Wakayama Medical University Hospital (WMUH).

Of the patients, 126 underwent OG and 639 underwent LG. Patients underwent LG as part of a clinical trial (UMIN000025029). The remaining 20 patients received RG. We started RG in 2017 as part of a phase II trial (UMIN000027969). In our institute, laparoscopic and RG is adopted for all GC patients in whom curative gastrectomy is applicable.

To compare short-term surgical outcomes, all consecutive patients who underwent laparoscopic and RG during this period were included in this retrospective study.

Tumor stage was classified by the International Union Against Cancer tumor-node-metastasis (TNM) criteria, Eighth Edition.^[12]^ Grades higher than Clavien–Dindo grade 2 were defined as clinically significant perioperative complications.^[13]^ POPF higher than grade 2 (requiring pharmacological treatment with drugs) were regarded as clinically significant.

### 2.2 Surgical procedures

#### 2.2.1 Laparoscopic gastrectomy

**Figure 1:**
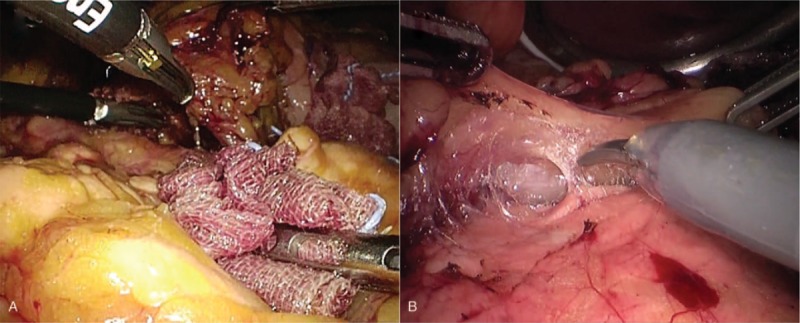
Surgical view during dissection of suprapancreatic lymph nodes. (A) Efficient posteroinferior compression of the pancreas with gauze by the assisting forceps allows effective visual development for a limited small surgical site during laparoscopic lymphadenectomy. (B) Compression of the pancreas with the assisting forceps is not necessary during robotic lymphadenectomy using articulating forceps.

Details of the LG procedures performed at WMUH have been previously described (Supplemental Digital Content 1).^[14,15]^ The basic extent of lymph node dissection in the present series was D1+ or D2.^[16]^ The greater omentum was resected up to the inferior portion of the spleen using laparoscopic USAD, the harmonic scalpel (Ethicon Endo-Surgery, Cincinnati, OH). The left gastroepiploic vessels were dissected at the point before the first branch (nos. 4d, 4sb). After completion of omentectomy, the root of the right gastroepiploic vein and artery were isolated and transected (no. 6). The root of the right gastric artery was isolated in the hepatoduodenal ligament and transected (no. 5). The lesser omentum along the liver edge to the esophagogastric junction was resected. The perigastric lymph nodes were dissected along the upper lesser curvature up to the esophagogastric junction (nos. 1 and 3). For laparoscopic D1+ lymphadenectomy, the lymph nodes around the celiac trunk (no. 9) were dissected, and the root of the left gastric vein and artery were isolated and transected using USAD (no. 7). Following that, the lymph nodes along the common hepatic artery were then dissected (no. 8a). For laparoscopic D2 lymph node dissection, the lymph nodes along the proper hepatic artery (no. 12a) and along the splenic artery (no. 11) were also dissected. Lymph node dissection was completed intracorporeally. In laparoscopic dissection of suprapancreatic lymph nodes, postinferior efficient compression of the pancreas with gauze from the assisting forceps allowed effective visual development in a limited small surgical site (Fig. 1A).

#### 2.2.2 Robotic gastrectomy

All RG procedures were performed using da Vinci S or Si Surgical System (Intuitive, Sunnyvale, CA) with 4 articulating robotic arms; a central arm for a 30° rigid endoscope, a first arm for monopolar scissors, a second arm for fenestrated bipolar forceps, and a third arm for Cadiere forceps.^[17–21]^ An additional port for assisting forceps was made in the right umbilical level. As robotic USAD does not have wrist-like motion, and therefore no robotic articulated function, we did not use it. RG procedures did not differ from the LG procedure with D1+ or D2 lymph node dissection as described above. Unlike LG procedure, however, compression of the pancreas with gauze from the assisting forceps was not necessary during robotic dissection of peripancreatic lymph nodes. In RG using articulating forceps, lymphadenectomy without touching the pancreas was possible (Fig. 1B).

### 2.3 Statistical examinations

SPSS version 24.0 (SPSS, Chicago, IL) was used for all statistical analyses. Quantitative results were expressed as medians and ranges. Statistical comparisons between both groups were performed using chi-squared statistics, Fisher exact test, and Mann–Whitney *U* test. A *P* < .05 was considered to be significant.

## 3 Results

### 3.1 Clinicopathological characteristics of the patients

Patient characteristics are shown in Table 1. Of the 659 patients, 639 underwent LG, and the remaining 20 patients underwent RG. There were no differences between groups in age, male to female ratio, body mass index, or distribution of TNM stage.

**Table 1:**
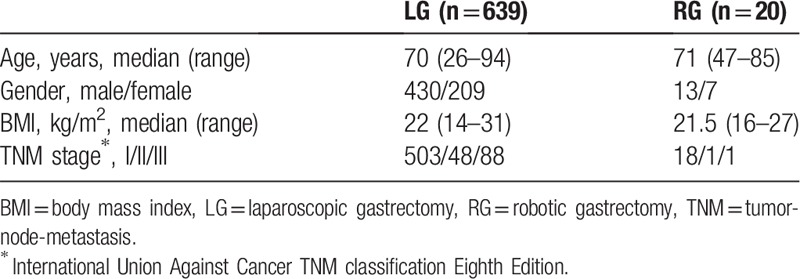
Clinicopathological patient characteristics.

### 3.2 Surgical results and clinical data

All 659 patients underwent curative resection. Surgical results were also stratified into the 2 surgical groups (Table 2). There were no differences between the groups in terms of subtotal gastrectomy to total gastrectomy ratio and range of lymphadenectomy. Duration of surgery was significantly longer in the RG group (386.5 min) than in the LG group (248 min) (*P* = .001). However, intraoperative blood loss was significantly less in the RG group (32.5 mL) than in the LG group (60 mL) (*P* = .001). The number of retrieved lymph nodes was significantly larger in the RG group (33) than in the LG group (24) (*P* = .017).

**Table 2:**
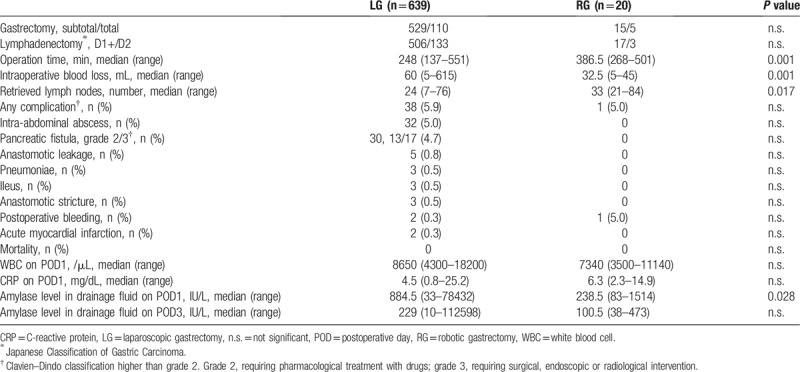
Surgical results and clinical data.

There was no significant difference in the overall incidence of postoperative complications (higher than Clavien–Dindo grade 2) in the LG group and in the RG group (5.9% vs. 5.0%). In our consecutive series, POPF, intra-abdominal abscess, and anastomotic leakage were not found in the RG group, but postoperative bleeding requiring interventional catheter embolization occurred in 1 patient (grade 3a). In this patient, although POPF was absent, intra-abdominal bleeding was detected on postoperative day (POD) 14. The bleeding was caused by the hemoclip dropping out, which ligated a left gastric artery. After interventional catheter treatment, this patient recovered fully. In contrast, POPF was found in 4.7% of the patients in the LG group. Thirteen patients were grade 2 and 17 patients were grade 3. Mortality rate was zero in both groups.

There were no differences in white blood cell count and CRP levels on POD 1 between the groups. Amylase levels in drainage fluid on POD 1 were significantly lower in the RG group (238.5 IU/L) than in the LG group (884.5 IU/L) (*P* = .028). However, these values on POD 3 were no significantly different (100.5 IU/L vs. 229 IU/L).

## 4 Discussion

We compared the surgical results of RG and LG for GC in 659 patients. Although duration of surgery in the RG group was longer than in the LG group, bleeding was significantly lesser in the RG group. Other studies also report long duration of surgery in RG.^[9,10,17–19]^ Considering the complexity of the RG operative procedures, to a certain degree, length of surgery cannot be helped. Further improvement in surgical skills is therefore necessary to shorten RG operation time.

Although there was less bleeding in RG than in LG in our study, whether RG has less bleeding than LG remains controversial.^[9,10,17–20]^ We propose that the advantages of robotic surgery, such as high-resolution monitor with three-dimensional vision, tremor filtering, and articulated forceps, can decrease intraoperative bleeding.

In our results, the rates of complication incidence were comparable between the RG and LG groups. We postulate that RG is a safe and feasible alternative to LG with regard to short-term surgical outcomes. In the LG group, POPF was found in 4.7% of the patients; but no POFP was found in the RG group. Also, amylase levels in drainage fluid on POD 1 were significantly lower in the RG group than in the LG group. Our RG procedure, which avoids contact with the pancreas, may result in reduced instances of POPF.

One cause of POPF may be the compression of the pancreas by the assisting forceps during lymphadenectomy of peripancreatic lymph nodes, particularly infrapyloric lymph node (no. 6), around the common hepatic artery (no. 8a), around the celiac trunk (no. 9), and along the splenic artery (no. 11).^[16]^ In laparoscopic lymphadenectomy, efficient compression of the pancreas with gauze by the assisting forceps allows effective visualization in a limited small surgical site. The power, direction, and general activity of the assistance, however, vary. We and others have reported occurrences of POPF in LG.^[9,10,14,18,21]^ On the other hand, the articulated forceps of the robot make it easier to access the suprapancreatic area. Unlike in LG, robotic lymphadenectomy without touching the pancreas is possible if RG technique is standardized. A “solo surgery” may be associated with reduced POPF.

Direct injury to process of pancreas head (PPH) during dissection of the infrapyloric lymph nodes is also thought to be a cause of POPF.^[22]^ PPH is covered by the mesoduodenum, which is adjacent to the pylorus.^[22]^ During laparoscopic dissection of the infrapyloric lymph nodes, the deep adipose tissue covering the surface of PPH can lead to misidentification of its presence. In patients undergoing robotic lymphadenectomy, however, the dissection layer between the adipose tissue and the PPH was clear, as shown in our video. We believe that the advantages of robotic surgery may reduce misidentification of PPH as adipose tissue.

Thermal injuries from electric dissections may also have an important role in causing POPF.^[23]^ We and others have used laparoscopic USAD during laparoscopic lymphadenectomy.^[9,10,14,18,23]^ High-power ultrasonic dissection may result in considerable heat production that could damage surrounding tissue. Thermal damage to the pancreas due to ultrasonic dissection during LG is therefore a possibility. We did not use robotic USAD during RG, however, because it does not have articulated function. Not using USAD in our procedure may be another reason for there being no occurrence of POPF in patients who underwent RG.

This study has several limitations. It was a single-center, retrospective study without randomized controlled trial (RCT), and comprised a small sample size. The imbalance between robotic and laparoscopic groups (20 vs. 639) could introduce bias in the statistical analysis and reduce the power of the study. Additionally, patients were allocated to the 2 groups according to the sequential nature of the surgery. There were differences in the length of follow-up and unclear inclusion criteria and indications for the 2 procedures, adding bias to the study. Finally, long-term oncological outcomes have not been investigated. From April 2018, we therefore started a prospective RCT to evaluate the short and long-term outcomes of GC patients treated with RG and LG (UMIN000031536).^[24]^

In conclusion, RG is a feasible and safe procedure for GC regarding short-term surgical outcomes. Our robotic procedure, which does not touch the pancreas, may allow for decreased incidence of POPF. The benefits of RG will be further validated in the ongoing prospective RCT.

## Author contributions

Study concept and design: Toshiyasu Ojima, Hiroki Yamaue.

Acquisition of data: Toshiyasu Ojima, Masaki Nakamura, Mikihito Nakamori, Keiji Hayata, Shimpei Maruoka.

Analysis and interpretation of data: Toshiyasu Ojima, Masaki Nakamura, Masahiro Katsuda.

Drafting of the manuscript: Toshiyasu Ojima, Masaki Nakamura, Masahiro Katsuda.

Critical revision of the manuscript for important intellectual content: Hiroki Yamaue. Statistical analysis: Toshiyasu Ojima, Keiji Hayata, Hiroki Yamaue.

Administrative, technical, and material support: Masaki Nakamura, Mikihito Nakamori, Keiji Hayata, Masahiro Katsuda, Hiroki Yamaue.

**Conceptualization:** Toshiyasu Ojima, Hiroki Yamaue.

**Data curation:** Toshiyasu Ojima, Masaki Nakamura, Mikihito Nakamori, Keiji Hayata, Shimpei Maruoka.

**Formal analysis:** Toshiyasu Ojima, Masaki Nakamura, Masahiro Katsuda.

**Investigation:** Toshiyasu Ojima, Masaki Nakamura, Mikihito Nakamori, Keiji Hayata, Masahiro Katsuda.

**Methodology:** Mikihito Nakamori.

**Project administration:** Masahiro Katsuda, Hiroki Yamaue.

**Supervision:** Hiroki Yamaue.

**Visualization:** Toshiyasu Ojima.

**Writing – Original Draft:** Toshiyasu Ojima.

**Writing – Review & Editing:** Hiroki Yamaue.

## Supplementary Material

Supplemental Digital Content
